# High Power and Large-Energy Pulse Generation in an Erbium-Doped Fiber Laser by a Ferromagnetic Insulator-Cr_2_Si_2_Te_6_ Saturable Absorber

**DOI:** 10.3390/nano12030564

**Published:** 2022-02-07

**Authors:** Zhifeng Hong, Xiwen Jiang, Meixia Zhang, Huanian Zhang, Xiaojuan Liu

**Affiliations:** School of Physics and Optoelectronic Engineering, Shandong University of Technology, Zibo 255049, China; hongzf0212@163.com (Z.H.); jiangxiwen20211010@163.com (X.J.); meixia0524@163.com (M.Z.); huanian_zhang@163.com (H.Z.)

**Keywords:** Cr_2_Si_2_Te_6_ saturable absorber, erbium-doped fiber, large-energy pulse generation, mode-locked fiber lasers

## Abstract

Large-energy mode-locked fiber lasers are extensively studied due to their indispensable use in various fields and applications. Recently, ferromagnetic insulators have attracted tremendous research interest in ultra-fast photonics because of their unique ferromagnetic properties and typical layered structure. In our work, Cr_2_Si_2_Te_6_ nanosheets are prepared and utilized as a saturable absorber (SA) in a large-energy mode-locked erbium-doped fiber (EDF) laser. With a total cavity length of 240 m, a stable mode-locked operation characterized by maximum pulse energy as high as 244.76 nJ with a repetition rate of 847.64 kHz is achieved. When the cavity length is extended to 390 m, the output maximum pulse energy is successfully scaled up to 325.50 nJ. To our knowledge, this is the largest pulse energy and highest output power level to be achieved in mode-locked fiber lasers by two-dimensional (2D) material saturable absorbers (SAs) so far. This work not only makes a forward step to the investigation of the generation of large-energy pulses in mode-locked fiber lasers but also fully proves that the ferromagnetic insulator-Cr_2_Si_2_Te_6_ possesses an excellent nonlinear absorption property, antioxidant capacity in ambient conditions, as well as outstanding thermal stability, which enriches our insight into 2D materials.

## 1. Introduction

Reliable, ultrafast fiber lasers are one of the most sought-after applications due to the advantages of their miniaturization, compact structure, and high reliability [1,2,3,4,5,6,7,8,9,10,11,12,13,14]. Among them, large-energy mode-locked fiber lasers have attracted more and more attention due to their indispensable applications in industrial material processing, medical treatment, scientific research, and other fields. Various methods were previously adopted to scale up the pulse energy. Involved techniques, such as increasing the pump power, optimizing the splitting ratio of the optical coupler (OC), and optimizing the saturable absorber’s (SAs) parameters, as well as cavity design, are most often applied. In 2014, Fu et al. realized a mode-locked operation by a graphene SA, which achieved maximum pulse energy of 16.2 nJ at a center wavelength of 1908 nm [15]. In 2018, Xu et al. implemented a mode-locked erbium-doped fiber (EDF) laser using a Bi_2_Se_3_-based SA, in which maximum pulse energy of 17.2 nJ was obtained [16]. In 2019, Ma et al. achieved a stable mode-locked EDF laser with a maximum pulse energy of 130.49 nJ using a MoS_2_ SA [17]. The following year, Li et al. established a high-energy EDF laser based on a VSe_2_ SA, and maximum pulse energy of 25.57 nJ was obtained [18].

From the articles mentioned above, on the other hand, it is seen that SAs are core devices to sharpen the pulse in the temporal domain, and the research on various new SAs has greatly promoted the development of pulsed lasers in the last decade. Except for the traditional semiconductor saturable absorber mirrors (SESAMs), two-dimensional (2D) materials [19,20,21,22,23,24,25,26,27], quantum dots [28,29], metal nanoparticles [30,31,32,33], and other new SAs have been intensively studied. Furthermore, after graphene was found to have saturable absorption properties and used as SA for mode-locked lasers in 2009 [34], a wide range of 2D materials such as transition metal dichalcogenides (TMDs) [1,2,3,4,5,6,7,8,9,10,11,12,13], topological insulators (TIs) [19,20,21,22], black phosphorus (BP) [23,24], MXenes [25,26,27], were shown to have great potential in the construction of high-performance and new functional optoelectronic devices [1,2,3,4,5,6,7,8,9,10,11,12,13,14,15,16,17,18,19,20,21,22,23,24,25,26,27]. Still, researchers are constantly paying tremendous attention to exploring SA materials with admirable features of high nonlinearity, high power handling ability, fast response time, wide absorption band, ambient atmospheric stability, etc., and in return, such efforts motivate the significant development of ultrafast fiber lasers. Fortunately, ferromagnetic insulators are found to possess superb ferromagnetic properties and a typical layered structure, hence arousing great research enthusiasm in the field of ultrafast photonics. In 2019, Guo et al. prepared a ferromagnetic insulator-Cr_2_Ge_2_Te_6_ polyvinyl alcohol (PVA) thin-film. By applying it to an EDF laser as SA, stable mode-locked pulses with a pulse duration of 550 fs were achieved [35]. Subsequently, Zhao et al. reported a Cr_2_Ge_2_Te_6_-SA based dark-bright soliton fiber laser with a maximum output power of 13.614 mW [36]. On the basis of the works above, ferromagnetic insulator-Cr_2_Ge_2_Te_6_ was proven to be a capable SA for ultrafast fiber lasers. Using the method of analogy, Cr_2_Si_2_Te_6_, another ferromagnetic insulator, is inferred to have similar nonlinear saturable absorption properties. Regarding aspects of Cr_2_Si_2_Te_6_, the material exhibits a typical layered, nearly 2D-hexagonal structure and possesses special transport magnetic, optical, and calculated electronic properties. Especially, the indirect bandgap value of Cr_2_Si_2_Te_6_ is 0.6 eV, which is narrower than that of Cr_2_Ge_2_Te_6_ (0.7 eV) [37]. Additionally, its nonlinear absorption coefficient is approximately equal to that of TMD. Based on the comparison, Cr_2_Si_2_Te_6_ is expected to show excellent absorption properties in the wavelength range from visible to mid infrared [38,39]. However, to the author’s best knowledge, the study of pulse lasers utilizing Cr_2_Si_2_Te_6_ as SAs is rare so far.

In this work, Cr_2_Si_2_Te_6_ nanosheets are prepared by ultrasonic-assisted liquid-phase exfoliation (LPE) method. The saturation intensity and modulation depth of the Cr_2_Si_2_Te_6_-PVA modulator are 10.4 MW/cm^2^ and 8.6%, respectively. In the large-energy mode-locked EDF laser experiment, the cavity length is originally set as 240 m, and a stable mode-locked operation is achieved. The obtained largest pulse energy is 244.76 nJ, with a central wavelength of 1561.13 nm and a repetition rate of 847.64 kHz. In order to increase the output pulse energy, the pulse repetition rate is reduced by simply successively increasing the cavity length. As expected, when the cavity length is extended to 390 m, the maximum pulse energy is successfully increased up to 325.50 nJ. To our knowledge, this is the largest pulse energy as well as the highest output power level reported in mode-locked fiber lasers modulated by 2D material SAs so far. Besides, the experimental results reveal the high stability of the system both in a large pump power range (as high as 300–1100 mW) and a large cavity length range (240–390 m). Our experiment provides references for further research on large-energy mode-locked fiber lasers as well as enriches the insight into 2D materials.

## 2. Fabrication and Characterization of Cr_2_Si_2_Te_6_ SA

The Cr_2_Si_2_Te_6_-PVA thin film modulator is prepared using an ultrasonic-assisted liquid-phase exfoliation (LPE) method. First, 1 g Cr_2_Si_2_Te_6_ powder is added to 60 mL ethanol (30%) to prepare a Cr_2_Si_2_Te_6_ dispersion solution. In detail, the mixture is treated under bath sonication conditions for 10 h to obtain Cr_2_Si_2_Te_6_ dispersion. The as-prepared Cr_2_Si_2_Te_6_ solution subsequently undergoes a high-speed centrifugation process at 1500 rpm for 30 min to obtain nanosheets. The supernatant liquor is collected for characterization and SA fabrication. Second, the Cr_2_Si_2_Te_6_ supernatant liquor is mixed with 5 wt.% PVA solution in a volume ratio of 1:1. Then, the mixture is treated using an ultrasonic bath process for another 5 h to obtain uniformity. As a result, the Cr_2_Si_2_Te_6_-PVA dispersion is obtained. In the process, PVA is used as the polymer matrix both to form film to host the Cr_2_Si_2_Te_6_ and to avoid its oxidation. Finally, 100 μL polymer mixture is spin-coated on a sapphire substrate and, after slow evaporation in an oven at 30 °C for 24 h, a filmy freestanding Cr_2_Si_2_Te_6_-PVA composite is prepared. For the commonly used sandwich-structure mode-locker, a 0.1 cm × 0.1 cm film is cut off and transferred onto the end faces of fiber connectors, and the proposed Cr_2_Si_2_Te_6_ SA anticipates ultrafast laser operation.

**Figure 1:**
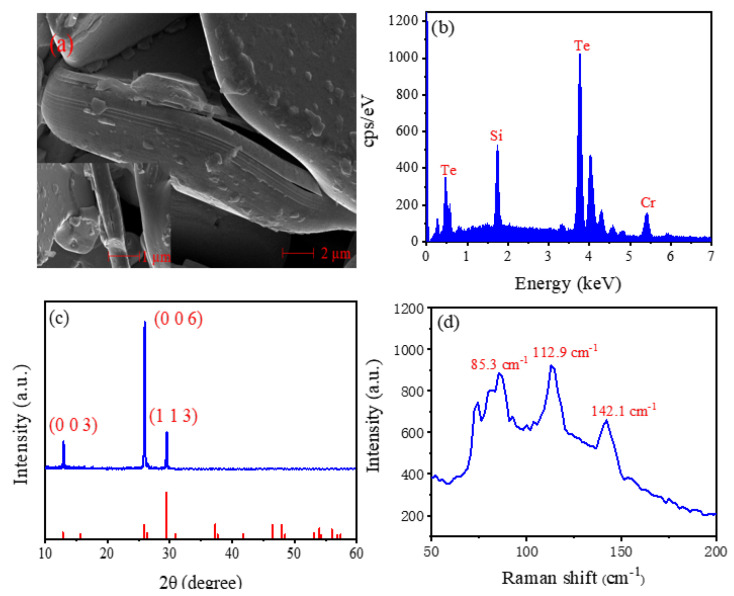
Crystal structure of the Cr_2_Si_2_Te_6_ nanosheets. (**a**) SEM image with 2 µm resolution. Inset: SEM image with 1 µm resolution. (**b**) EDX spectroscopy. (**c**) XRD. (**d**) Raman spectrum.

The morphological structure of the prepared Cr_2_Si_2_Te_6_ nanosheets is analyzed by scanning electron microscopy (SEM, S4800, HITACHI, Tokyo, Japan). Figure 1 show an SEM image at the resolution of 2 µm, and the well-layered structures are obvious. Figure 1b depict the corresponding energy-dispersive X-ray (EDX, S4800, HITACHI, Tokyo, Japan) spectroscopy, which reveals the typical peaks of Cr, Si, and Te. The X-ray diffraction (XRD, AXS D8 Advance, Bruker, Billerica, MA, USA) pattern of the Cr_2_Si_2_Te_6_ nanosheets is shown in Figure 1c, from which a high diffraction peak in the (006) plane can be seen, indicating the excellent crystallization properties of Cr_2_Si_2_Te_6_ nanosheets. The Raman spectra of Cr_2_Si_2_Te_6_ nanosheets are tested, and the data are illustrated in Figure 1d. As can be seen, the three symbolic scattered peaks of Cr_2_Si_2_Te_6_ are acquired at 85.3 cm^−1^, 112.9 cm^−1^, and 142.1 cm^−1^, respectively. The results reveal that pure Cr_2_Si_2_Te_6_ nanosheets with well-layered structures are prepared.

**Figure 2:**
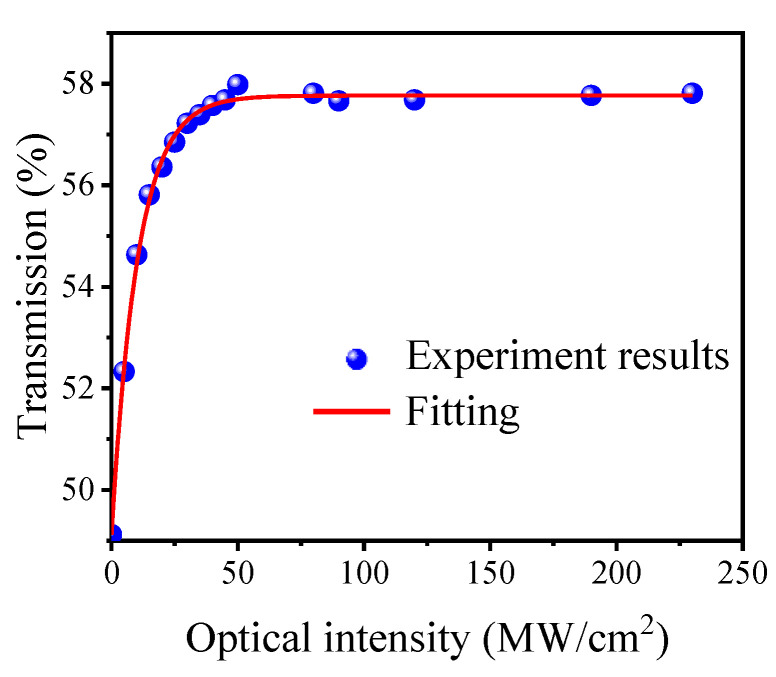
Saturable absorption curve of the Cr_2_Si_2_Te_6_-PVA film.

The nonlinear absorption characteristic of the Cr_2_Si_2_Te_6_-PVA film is investigated by the commonly used balanced twin detector measurement scheme. A mode-locked EDF laser based on a nonlinear polarization rotation regime is used as the pump source, and the center wavelength, repetition rate, and pulse duration are 1581 nm, 33.6 MHz, and 560 fs, respectively. The intensity-dependent nonlinear normalized saturable absorption curve is presented as blue dots in Figure 2. Using a fitting analysis, presented as a red line, the data agree well with the curve of the saturable absorption formula:

(1) $$T\left(I\right)=1-T_{ns}-\Delta \exp\left(-\frac{I}{I_{sat}}\right),$$

where *T(I)* and Δ are the transmission and the modulation depth. *I* and *I_sat_* are the input laser intensity and the saturation intensity. *T_ns_* is the nonsaturable loss. According to the fitting analysis, the saturation intensity and modulation depth of the prepared Cr_2_Si_2_Te_6_ nanosheets are 10.4 MW/cm^2^ and 8.6%, respectively. These results provide a good understanding of the nonlinear optical properties of the prepared Cr_2_Si_2_Te_6_ nanosheets and confirm that the filmy Cr_2_Si_2_Te_6_ SA could be applied in fiber lasers for passive mode-locking due to the evident saturable absorption at 1550 nm.

## 3. Experimental Setup

**Figure 3:**
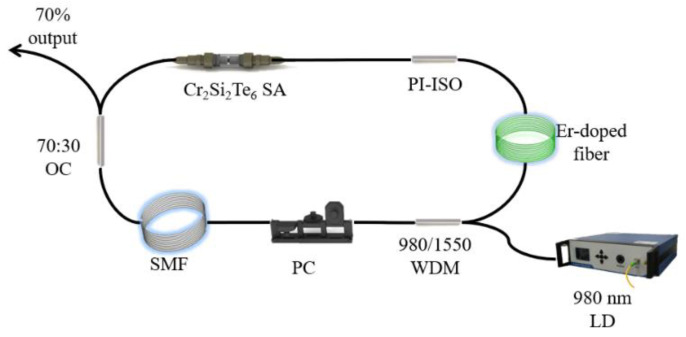
Experimental setup of the large-energy mode-locked EDF laser based on Cr_2_Si_2_Te_6_ SA.

The high power large-energy mode-locked EDF laser based on Cr_2_Si_2_Te_6_-SA is schematically depicted in Figure 3. A piece of 10-m long EDF is adopted as the gain medium. A 980 nm laser diode (LD) with a maximum output power of 1250 mW is employed as a pump source, which is coupled into the ring cavity through a 980/1550 nm wavelength division multiplexer (WDM). A polarization independent isolator (PI-ISO) is utilized to guarantee the unidirectional transmission of the light. An in-line polarization controller (PC) is used to fine tune the polarization state of the cavity. As high as 70% of the laser power is extracted out of the cavity by an optical coupler (OC). The Cr_2_Si_2_Te_6_-SA is inserted between the PI-ISO and the OC to enable the mode-locked operation. The dispersion coefficients of EDF and single-mode fiber (SMF) are −18 ps/nm/km and 17 ps/nm/km, respectively. Considering that the four pieces of SMFs adopted in the proposed experiment are 230, 285, 325, and 380 m long, the net dispersions of the ring cavity are −4.77, −6.11, −7.02, and −8.21 ps^2^, respectively. The output signal is monitored by a digital oscilloscope (OSC) (Wavesurfer 3054, LeCroy, Teledyne, USA) with a 3-GHz photo-detector (PD 03), an optical spectrum analyzer (OSA) (AQ6370B, Yokogawa, Tokyo, Japan), and a radio frequency spectrum analyzer (FPC1000, Rohde & Schwarz, Jena, Germany), while the average output power is measured by a power meter (PM3, Molectron, Barrington, NJ, USA).

## 4. Experimental Results and Discussion

At the beginning of the experiment, the cavity length is originally set as 240 m. Under the conditions of Cr_2_Si_2_Te_6_-SA being absent in the cavity, no mode-locked phenomenon is observed no matter how the pump power and PC are tuned in the whole range. Then, the prepared Cr_2_Si_2_Te_6_-PVA SA is inserted into the laser cavity. By gradually and carefully increasing the pump power and optimizing the polarization state of the cavity simultaneously, a stable mode-locked operation is initiated when the pump power is increased up to 300 mW. This process confirms the fact that the mode-locked operation is aroused by the modulation of the Cr_2_Si_2_Te_6_ SA. The laser can operate in a stable mode-locked regime in the power range of 300~1100 mW. No Q-switched operation, which is easier obtained in short cavities [3,7,22,40], is detected in the experimental duration.

**Figure 4:**
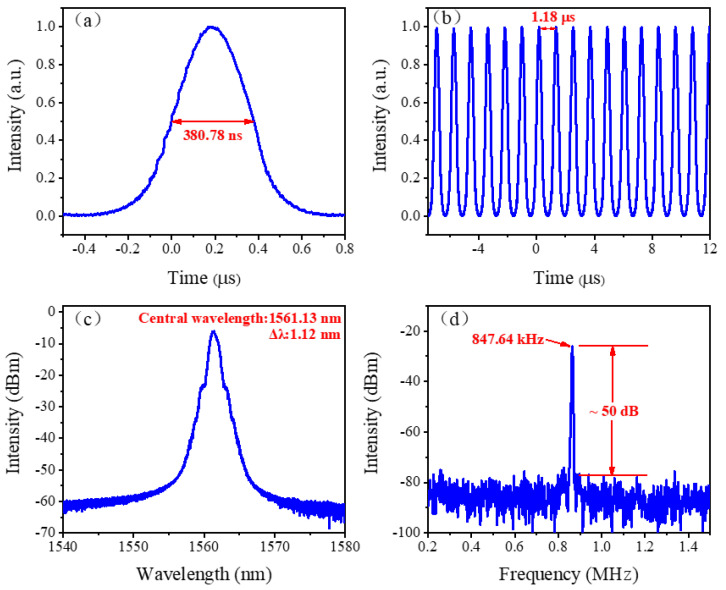
Mode-locked results of Cr_2_S_i2_Te_6_ SA thin film with a cavity length of 240 m. (**a**) Single pulse envelope. (**b**) Pulse trains. (**c**) Optical spectrum. (**d**) RF spectrum.

At the pump power of 1100 mW, a single-shot pulse envelope, as well as the corresponding pulse sequence, is recorded by digital oscilloscope and shown in Figure 4a,b, respectively. The pulse duration and the pulse interval are 380.78 ns and 1.18 μs. As is well known, the repetition rate of the Q switched pulses generally increases with the increase of pump power. However, the present experiment and the experiments demonstrated subsequently display that the repetition rate of the output pulses remains constant and matches the cavity length according to the following formula:

(2) $$T=\frac{\mu L}{c},$$

where *T*, *μ*, *L*, and *c* are the pulse interval, refractive index, cavity length, and the velocity of light, respectively. This denoted the relationship between the cavity length and pulse interval in mode-locked operation. Therefore, it is concluded that although the obtained pulse duration is a submicrosecond order of magnitude, the operation is in a mode-locked regime [41]. Furthermore, in order to rule out the possibility of noise-like pulses, an optical autocorrelator (Femtochrome FR-103 XL, Berkeley, CA, USA) is utilized to test the pulse durations of the present and the subsequent cavities. No pulse information is observed, which confirms that the pulse durations measured by the digital oscilloscope are accurate. The large pulse duration might be introduced by the large dispersion of the system, which is similar to the results of others [42,43].

The optical spectrum of the mode-locked pulse is shown in Figure 4c. The spectrum is centered at 1561.13 nm with a 3 dB bandwidth of about 1.12 nm. Considering that the net dispersion of the system is negative, conventional solitons with Kelly sidebands distributing symmetrically about the central wavelength are expected. However, as the output spectrum presented, no obvious Kelly sideband is observed. The results indicate that the pulse energy is highly concentrated in the proposed regime.

As a general indicator used to characterize the stability of a mode-locked fiber laser, the single-to-noise ratio (SNR) represents the contrast between detected pulses and background noise signals. To obtain the SNR, the radio frequency (RF) spectrum of the mode-locked pulses is scanned. As depicted in Figure 4d, the SNR is measured to be about 50 dB, and the fundamental frequency repetition rate is 847.64 kHz. The former exhibits the high stability of the system, and the latter indicates that the repetition rate is consistent with the pulse interval (*f* = 1/*T*), which further confirms the mode-locked state of the system.

**Figure 5:**
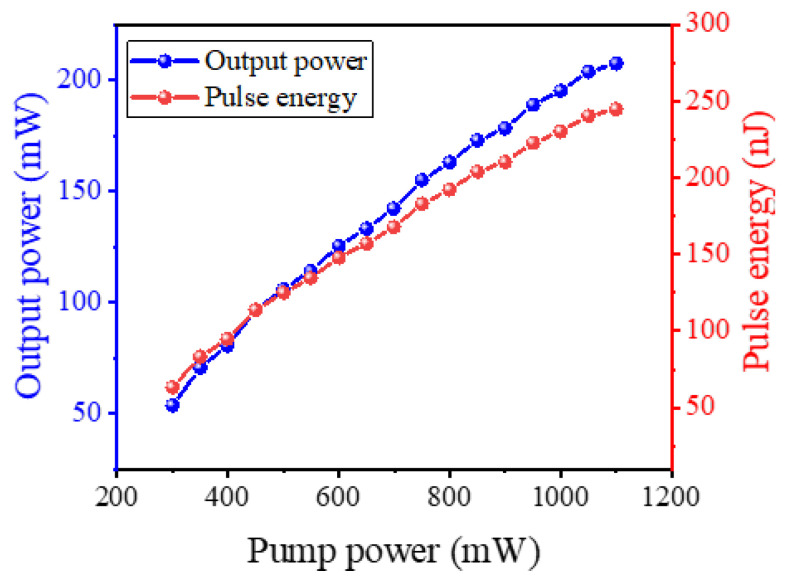
Output power and pulse energy versus the pump power.

Figure 5 depict the output power and pulse energy as functions of pump power. Both the output power and pulse energy almost increase linearly with the pump power. When the pump power is 1100 mW, the obtained largest output power is 207.47 mW, corresponding to an optical-to-optical conversion efficiency of 18.9%. It is worth emphasizing that the obtained maximum pulse energy is 244.76 nJ, which is significantly greater than the conventional mode-locked operations. The results indicate high stability in a large pump power range.

**Figure 6:**
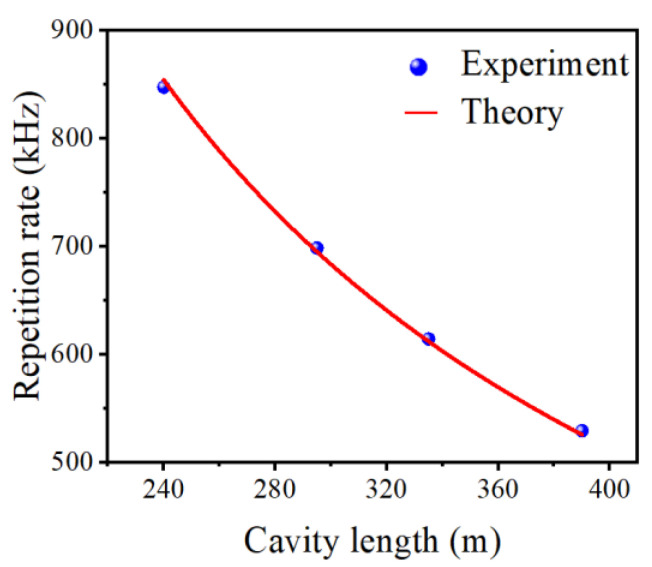
Variations of pulse repetition rate with the cavity length.

In order to obtain large-energy pulse generation, according to (2), the proposal in our work is to reduce the repetition rate by extending the SMF in the laser cavity. As a result, the accumulated power in the cavity will be distributed to fewer mode-locked pulses, and hence the energy for each pulse will be enlarged. In the actual implementation process, four SMFs with lengths of 230, 285, 325, and 380 m are successively adopted. As shown in Figure 6, the fundamental frequency pulse repetition rate of the mode-locked fiber laser decreases with the extending of the SMFs, which is consistent with our deduction. Theoretically, the repetition rate (*f* = 1/*T* = $\frac{c}{\mu L}$) is inversely proportional to the cavity length, which is shown by the red curve in Figure 6. Apparently, the experiment data (blue dots) match well with the theoretical value. The slight deviation mainly comes from the inaccurate measurement of the fiber length and is negligible. Such results reveal high stability in a large cavity length range.

**Figure 7:**
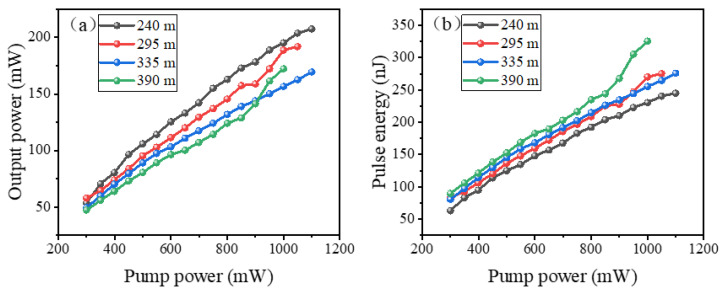
(**a**) Output power and (**b**) Pulse energy versus pump power of the four cavities with different lengths.

The variations of output power with pump power under the conditions of different cavity lengths are illustrated in Figure 7a. It is seen that, at the same pump power level, the average output power tends to decrease with the increase of the cavity length, and the highest output power is obtained by the shortest cavity. The reason is that the laser gain in the cavity is limited by the gain fiber with definite length as well as the pump power with a definite level. If the optimum cavity length is exceeded, the redundant SMF will absorb the laser oscillating in the cavity hence the total power decreases with the extending of the SMF. However, under the conditions of different cavity lengths, the pulse energy, as illustrated in Figure 7b, shows contrast results, i.e., the pulse energy increases with the extending of the SMF, and the largest pulse energy is achieved by the longest cavity. This can be explained by the relationship formula

(3) $$E_{\mathrm{pulse}}\times \nu_{\mathrm{pulse}}=P_{\mathrm{average}},$$

where *E*_pulse_, *ν*_pulse_ and *P*_average_ are the pulse energy, repetition rate, and output power of the mode-locked pulses. Considering that one of the dominant targets of our experiment is to obtain large-energy pulse generation in mode-locked fiber lasers, it is seen that on the premise of successful frequency reduction, although the output power is slightly decreased, the pulse energy can still be raised to a much higher level. Such a result fully confirms the feasibility of our proposal described above.

**Figure 8:**
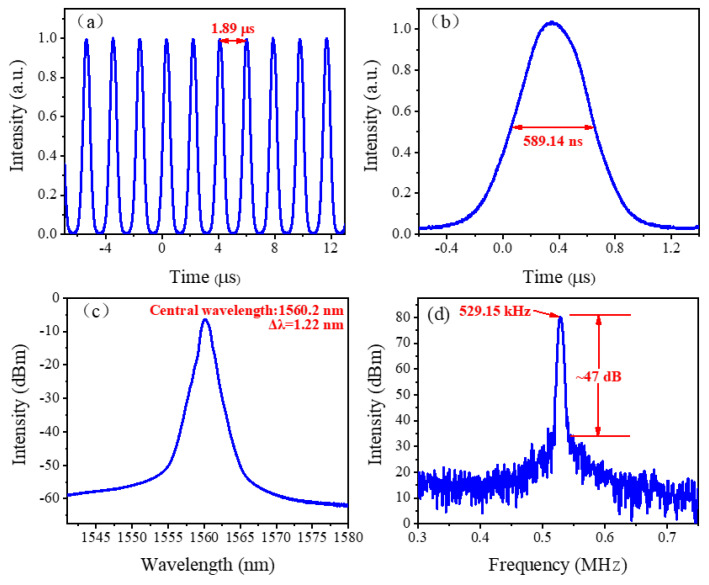
Mode-locked operation with a cavity length of 390 m. (**a**) Pulse trains. (**b**) Single pulse envelope. (**c**) Optical spectrum. (**d**) RF spectrum.

In the experiment, the largest pulse energy is achieved when the cavity length is 390 m. In detail, the pulse sequence and the single pulse envelope are exhibited in Figure 8a,b, respectively. It is presented that the pulse interval is 1.89 μs, and the pulse duration is 589.14 ns. The spectrum shown in Figure 8c is centered at 1560.20 nm with a 3 dB bandwidth of 1.22 nm. The measured SNR is 47 dB, and the fundamental frequency repetition rate is 529.15 kHz, which is exhibited in Figure 8d. The available maximum output power is 172.24 mW, so the largest pulse energy is calculated to be 325.50 nJ. During the process, the length of gain fiber remains unchanged, and when the pulse repetition rate is reduced from 847.64 kHz to 529.15 kHz by extending the length of SMF, the pulse energy is successfully scaled up from 244.76 nJ to 325.50 nJ. To our best knowledge, this is the largest pulse energy obtained in mode-locked fiber lasers modulated by 2D material SAs so far.

**Figure 9:**
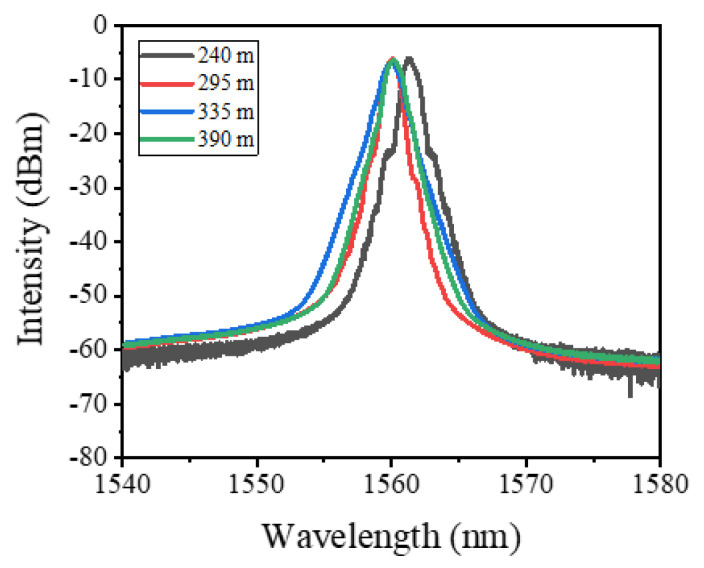
Optical spectra at the maximum output power level of the four cavities with different lengths.

The optical spectra of the four lasers with different cavity lengths are separately measured at the highest output power level. As depicted in Figure 9, it is seen that although the cavity lengths are different, there are no obvious changes in the optical spectra. The little shift between the spectrum output by the 240-m-long cavity and the other three spectra might be induced by the slight environmental changes because the time interval between the first experiment and the other three experiments is about three months.

For comparison, we summarize the performances of large-energy mode-locked lasers based on different 2D material SAs [16,17,18,42,43,44,45,46,47,48,49,50,51]. As listed in Table 1, it is seen that the pulse energy achieved in our work is remarkably superior to all the previous works, and in terms of output power, the result also belongs to the highest level. The experiment not only reveals the fact that Cr_2_Si_2_Te_6_-based SA is a strong competitor for mode-locked EDF lasers but also confirms the conclusion that our experimental regime is highly effective for generating large energy mode-locked lasers.

## 5. Conclusions

In summary, the Cr_2_Si_2_Te_6_-PVA sheets are prepared using an ultrasonic assisted LPE method. The saturation intensity and modulation depth of the prepared Cr_2_Si_2_Te_6_-PVA SA are 10.4 MW/cm^2^ and 8.6%, respectively. Then the modulator is used in the large-energy mode-locked EDF laser experiment. With the pulse repetition rate being successively reduced from 847.64 kHz to 529.15 kHz by extending the length of SMFs, the pulse energy is successfully scaled up from 244.76 nJ to 325.50 nJ, which is consistent with our proposal. There are no obvious changes in the optical spectra. The experimental results reveal the high stability of the system both in a large pump power range (as high as 300–1100 mW) and a large cavity length range (240–390 m). More importantly, the performance in terms of output power and pulse energy is remarkably superior to all the previous similar works. In the process, the ferromagnetic insulator-Cr_2_Si_2_Te_6_ exhibits excellent nonlinear absorption properties, antioxidant capacity in ambient conditions, as well as outstanding thermal stability, which are critical merits in practical applications. The work not only gives further insight into large-energy pulse generation in mode-locked fiber lasers but also enriches the exploration of 2D materials.

## Figures and Tables

**Table 1:** Comparison of large energy mode-locked lasers based on 2D SAs.

SA	Method	Modulation Depth (%)	Center Wavelength (nm)	Output Power (mW)	Pulse Energy (nJ)	Ref.
Graphene	LPE	—	1555	80	5.2	[42]
BP	LPE	12	1559.5	53	6	[43]
WS_2_	CVD	0.9	1562.5	19.8	90	[44]
InSe	LPE	13.7	—	11.96	20.4	[45]
MoS_2_	CVD	25.3	1564.6	122.77	130.49	[17]
MoSe_2_	LPE	5.4	1557.3	22.8	0.0067	[46]
WSe_2_	CVD	52.38	1562	30	0.51	[47]
Bi_2_Se_3_	LPE	—	1560.5	33.8	62.87	[16]
Bi_2_Se_3_	CVD	5.11	1561.9	185.3	171.3	[48]
Bi_2_Te_3_	PLD	6.2	1564	45.3	0.0154	[49]
VSe_2_	LPE	1.85	1565.7	53.21	25.57	[18]
Sb_2_Te_3_	PLD	7.42	1530	12	0.127	[50]
In_2_Se_3_	MSD	4.5	1565	83.2	2.03	[51]
Cr_2_Si_2_Te_6_	LPE	8.6	1560.2	172.24	325.50	Our work

LPE, liquid-phase exfoliation; CVD, Chemical Vapor Deposition; PLD, pulsed laser deposition; MSD, magnetron sputtering deposition.

## Data Availability

The data presented in this study are available on request from the authors.
